# Seroprevalence of *Borrelia burgdorferi*, *B. miyamotoi*, and Powassan Virus in Residents Bitten by *Ixodes* Ticks, Maine, USA

**DOI:** 10.3201/eid2504.180202

**Published:** 2019-04

**Authors:** Robert P. Smith, Susan P. Elias, Catherine E. Cavanaugh, Charles B. Lubelczyk, Eleanor H. Lacombe, Janna Brancato, Hester Doyle, Peter W. Rand, Gregory D. Ebel, Peter J. Krause

**Affiliations:** Maine Medical Center Research Institute, Scarborough, Maine, USA (R.P. Smith, Jr., S.P. Elias, C.B. Lubelczyk, E.H. Lacombe, P.W. Rand);; Lincoln Health, Damariscotta, Maine, USA (C.E. Cavanaugh);; Colorado State University, Fort Collins, Colorado, USA (G.D. Ebel);; Yale School of Public Health, New Haven, Connecticut, USA (J. Brancato, P.J. Krause);; Yale School of Medicine, New Haven (H. Doyle, P.J. Krause)

**Keywords:** Borrelia burgdorferi, Borrelia miyamotoi, bacteria, Ixodes scapularis, ticks, deer ticks, tick bites, Powassan virus, POWV, viruses, seroprevalence, vector-borne infections, zoonoses, meningitis/encephalitis, Maine, United States

## Abstract

We conducted a serosurvey of 230 persons in Maine, USA, who had been bitten by *Ixodes scapularis* or *I. cookei* ticks. We documented seropositivity for *Borrelia burgdorferi* (13.9%) and *B. miyamotoi* (2.6%), as well as a single equivocal result (0.4%) for Powassan encephalitis virus.

Reports of Lyme disease in Maine, USA, have increased from a few cases in the late 1980s to 1,848 cases in 2017 (*1*), coinciding with range expansion of *Ixodes scapularis* ticks over the past 3 decades (*2*). The Maine Center for Disease Control reported the first 2 cases of hard-tick relapsing fever caused by *Borrelia miyamotoi* during 2016 and an additional 6 cases during 2017 (*1*). Hard-tick relapsing fever typically manifests as a nonspecific febrile illness (*3**,**4*). Han et al. (*5*) found a *B. miyamotoi* infection prevalence of 3.7% in adult *I. scapularis* ticks in Maine, ≈10-fold less than that for *B. burgdorferi* infection (50%, range 32%–65%) (*6*).

Powassan virus (POWV) encephalitis can have devastating complications and has infected 10 residents of Maine during 2000–2017. There are 2 variants of POWV with distinct enzootic cycles and tick vectors. Lineage 1 is transmitted by *I. cookei* ticks and lineage 2, sometimes referred to as deer tick virus, is transmitted by *I*. *scapularis* ticks (*7*). Both lineages are present in Maine (*7*), but lineage 1 has a lesser risk for transmission because human bites by *I. cookei* ticks are infrequent (*8*). One fatal Maine case was demonstrated to be caused by lineage 2 POWV (*7*). Although POWV infection prevalence in Maine *I. scapularis* ticks is low (0.7%–1.8%) (*9*), frequent exposure to *I. scapularis* bites (*8*) and rapidity of POWV transmission (i.e., POWV can be transmitted to vertebrates after only 15 min from onset of the tick bite) (*10*) raise concern.

Our objective was to determine the seroprevalence of *B. burgdorferi*, *B. miyamotoi*, and POWV to clarify the frequency of exposure to each of these pathogens in resi-dents of Maine, USA, who had been bitten by *I. scapularis* or *I. cookei* ticks. We also anticipated that a serosurvey might provide evidence of asymptomatic POWV infection or self-limited illness in a few persons, as reported elsewhere (*11**,**12*).

## The Study

The Vector-Borne Disease Laboratory of the Maine Medical Center Research Institute provided a free, statewide tick identification service during 1989–2013 to monitor exposure to *I. scapularis* ticks during range expansion of this invasive vector of human and animal disease. Persons submitted ticks that they had removed from themselves, family members, and pets. As of 2014, 33,332 ticks representing 14 species were identified in Maine; *I. scapularis* ticks were predominant.

During 2014, we used our tick identification service database (*2*) to identify persons who had removed >1 attached *I. scapularis* or *I. cookei* tick(s) in the previous 5 years (2009–2013). We invited these persons to participate in a serosurvey to assess past exposure to *B. burgdorferi*, *B. miyamotoi*, and POWV. Family members who attended the clinic with these persons and who reported being bitten by ticks were also invited to participate. The study was approved by Maine Medical Center Institutional Review Board (Protocol #4222). Participants provided informed consent (assent for minors) and submitted 30 mL of blood. Blood was centrifuged at 3,500 rpm for 15 min. Serum aliquots were stored at −20°C and then shipped to testing laboratories.

Serologic testing for antibodies to *B. miyamotoi* was conducted at the laboratory of one of the authors (P.J.K.). An ELISA and confirmatory Western blot assay were used to detect serum reactivity to *B. miyamotoi* GlpQ protein (*13*). For the ELISA, serum samples were diluted 1:320 and a signal >3 SD above the mean of 3 *B. miyamotoi*–negative serum controls was considered positive for *B. miyamotoi* antibody. Serum samples were considered *B. miyamotoi* seropositive if ELISA IgG and Western blot IgG tests yielded positive results.

Serologic evidence of exposure to *B. burgdorferi* was detected by the standard 2-step ELISA and Western blot assay in the L2 Diagnostic Laboratory at Yale School of Medicine by one of the authors (H.D.). A reactive serum was defined as one that reacted to a dilution >1:100. All borderline or reactive serum was further characterized by Western blot immunoassay. Specimens were considered positive for *B. burgdorferi* exposure if the IgG immunoblot contained >5 of the 10 most common *B. burgdorferi*–associated bands (*14*).

Serologic testing for POWV was conducted by one of the authors (G.D.E.) by using a plaque-reduction neutralization test (PRNT) and a POWV–West Nile virus (WNV) chimeric virus (POWV–premembrane–envelope [prME]/WNV) assay as described (*15*). The specificity of the assay was determined by cross-neutralization studies, which demonstrated that antiserum raised against POWV efficiently neutralized chimeric POWV–prME/WNV but not WNV and that antiserum raised against WNV did not neutralize POWV–prME/WNV (*15*). Use of the chimeric POWV–prME/WNV assay virus enabled PRNT testing to be conducted on African green monkey kidney (Vero) cells according to standard procedures by using a 90% neutralization cutoff to be considered positive (*15*).

Of 230 enrolled persons, 190 were in our tick identification program database, and 40 were family members (Table 1). Among the 190 persons, 1 tick bite was from an *I. cookei* nymph, 13% of bites were from *I. scapularis* nymphs, and 86% of bites were from *I. scapularis* adult females. Engorgement of ticks ranged from slight (43%) to moderate (38%) to high (18%). Among the study population, 32 (13.9%) were seropositive for *B. burgdorferi*, 6 (2.6%) were seropositive for *B. miyamotoi*, and 2 (0.9%) were seropositive for both pathogens (Table 2). The serum of 1 person (0.4%) neutralized POWV at a titer of 1:20 and WNV at a titer of 1:10. We designated this serum as flavivirus positive. This person reported a history of neurologic illness for >1 year and a tick bite within the study year.

**Table 1:** Characteristics of residents bitten by blacklegged (deer) ticks (*Ixodes scapularis*) during 2009 and 2013 who participated in a serosurvey for antibodies against *Borrelia burgdorferi*, *B. miyamotoi*, and Powassan virus, Maine, USA, 2014*

Characteristic	No. (%) residents
Mailings and responses	
No. persons mailed	1,253
No. persons attending a clinic	230
No. database persons	190
No. persons from families of database persons	40
Clinic information	
Location	
Biddeford clinics: April 23 and 26, 21 towns	31 (13.5)
Ellsworth clinics: Apr 18 and 19, 34 towns	94 (40.9)
Rockland clinics: Apr 5 and 18, 21 towns	32 (13.9)
Scarborough clinics: Apr 10 and 12, 36 towns	73 (31.7)
Tick bite history and demographics of persons in database	
Year tick submitted	
2009	27 (14.2)
2010	41 (21.6)
2011	47 (24.7)
2012	37 (19.5)
2013	38 (20.0)
Tick species/stage	
*Ixodes cookei* nymph	1 (0.5)
*Ix. scapularis* female	164 (86.3)
*Ix. scapularis* nymph	25 (13.2)
Tick engorgement	
Slight	82 (43.2)
Moderate	73 (38.4)
	
Heavy	35 (18.4)
Age, y, at time of bite	
Adults, range 19–84	168 (88.4)
Children, range 6–18	22 (11.6)
Sex	
M	88 (46.3)
F	102 (53.7)
Demographics of all persons at time of clinic visit	
Age, y	
Adults, range 18–90	215 (93.0)
Children, range 8–17	15 (7.0)
Race	
Not reporting	3 (1.3)
American Indian/Alaska Native	0 (0.0)
Asian	2 (0.9)
Black or African American	0 (0.0)
Hispanic or Latino	1 (0.4)
Native Hawaiian/Pacific Islander	0 (0.0)
White	224 (98.7)
Sex	
M	107 (46.5)
F	123 (53.5)

*Database persons refers to tick-bitten persons who had submitted their ticks to a tick identification program in Maine. Family of database persons were database person family members who reported being bitten by a blacklegged tick during 2009–2013.

**Table 2:** Seropositivity of tick-bitten persons for *Borrelia burgdoferi*, *B. miyamotoi*, and Powassan virus, Maine, USA, 2014*

Pathogen, antibody test result	No. (%) database persons, n = 190	No. (%) family of database persons, n = 40	No. (%) total, n = 230
*B. burgdorferi*			
Positive	26 (13.7)	6 (15.0)	32 (13.9)
Negative	164 (86.3)	34 (85.0)	198 (86.1)
*B. miyamotoi*			
Positive	4 (2.1)	2 (5.0)	6 (2.6)
Negative	186 (97.9)	38 (95.0)	224 (97.4)
*B. burgdorferi/B. miyamotoi*			
Positive	2 (1.1)	0	2 (0.9)
Negative	190 (98.9)	40 (100.0)	228 (99.1)
Powassan virus			
Positive	0	1 (2.5)	1 (0.4)
Negative	190 (100.0)	39 (97.5)	229 (99.6)

*Database persons refers to tick-bitten persons who had submitted their ticks to a tick identification program in Maine. Family of database persons were database person family members who reported being bitten by a blacklegged tick during 2009–2013.

## Conclusions

Among residents of southern Maine with a history of *I. scapularis* tick bites, the percentage who were seropositive for *B. burgdorferi* was 5 times greater than that for *B. miyamotoi* (13.9% vs. 2.6%) and 35 times greater than the percentage of deer ticks infected with POWV (0.4%). Because our study population consisted of persons bitten by *I. scapularis* ticks (with engorgement ranging from slight to high), we expect seroprevalence to be greater in this group than in that of the general population. The *B. burgdorferi* seroprevalence of 13.9% in our study population was ≈1.5 times higher than the seroprevalence of 9.4% reported by Krause et al. (*13*) in healthy residents of southern New England. In contrast, the *B. miyamotoi* seroprevalence of 2.1% was comparable to the seroprevalence of 1%–3.9% reported by Krause at al. (*4**,**13*).

Of 1,854 cases of infection with *Borrelia* spp. reported in Maine in 2017, a total of 1,848 were attributed to Lyme disease and only 6 (0.3%) were attributed to *B. miyamotoi* (*1*). On the basis of a seroprevalence of ≈2% in this study and that *B. miyamotoi* might be transmitted by all tick stages, we believe that this disease is underdiagnosed in Maine (*5*). Our population was identified by history of tick exposure, rather than by symptoms. Our results therefore represent the relative frequency of exposure to these different agents rather than risk for illness.

Although the sensitivity and specificity of the 2-tier antibody assay for *B. burgdorferi* is better validated than those of the *B. miyamotoi* and POWV assays, the sensitivity and specificity of these assays are good (*13**–**15*). Nonetheless, our findings might represent overestimates or underestimates of actual exposure to these agents because of false-positive or false-negative results. These data provide evidence that humans are exposed to *B. burgdorferi*, *B. miyamotoi*, and POWV in Maine and help define the prevalence of human infection caused by each of these tickborne pathogens.
